# The Role of Catalyst Support, Diluent and Co-Catalyst in Chromium-Mediated Heterogeneous Ethylene Trimerisation

**DOI:** 10.1007/s11244-018-0891-8

**Published:** 2018-01-16

**Authors:** M. J. Lamb, D. C. Apperley, M. J. Watson, P. W. Dyer

**Affiliations:** 1 Department of Chemistry, Durham University, South Road, Durham, DH1 3LE UK; 2 Department of Chemistry, Centre for Sustainable Chemical Processes, Durham University, South Road, Durham, DH1 3LE UK; 3 Johnson Matthey PLC, P. O. Box 1, Billingham, Cleveland TS23 1LB UK

**Keywords:** Heterogeneous catalysis, Chromium amide, 1-Hexene, Trimerisation, Oligomerisation, Polymerisation

## Abstract

Sequential treatment of a previously-calcined solid oxide support (i.e. SiO_2_, γ-Al_2_O_3_, or mixed SiO_2_–Al_2_O_3_) with solutions of Cr{N(SiMe_3_)_2_}_3_ (0.71 wt% Cr) and a Lewis acidic alkyl aluminium-based co-catalyst (15 molar equivalents) affords initiator systems active for the oligomerisation and/or polymerisation of ethylene. The influence of the oxide support, calcination temperature, co-catalyst, and reaction diluent on both the productivity and selectivity of the immobilised chromium initiator systems have been investigated, with the best performing combination (SiO_2−600_, modified methyl aluminoxane-12 {MMAO-12}, heptane) producing a mixture of hexenes (61 wt%; 79% 1-hexene), and polyethylene (16 wt%) with an activity of 2403 g g_Cr_^−1^ h^−1^. The observed product distribution is rationalised by two competing processes: trimerisation via a supported metallacycle-based mechanism and polymerisation through a classical Cossee-Arlman chain-growth pathway. This is supported by the indirect observation of two distinct chromium environments at the surface of the oxide support by a solid-state ^29^Si NMR spectroscopic study of the Cr{N(SiMe_3_)_2_}_x_/SiO_2−600_ pro-initiator.

**Electronic supplementary material:**

The online version of this article (10.1007/s11244-018-0891-8) contains supplementary material, which is available to authorized users.

## Introduction

Today’s market for short-chain linear α-olefins (LAOs) is so demanding that the traditional synthetic routes for their manufacture such as Ziegler- and SHOP-type oligomerisation processes, which give rise to statistical LAO product distributions, cannot keep pace [1]. As 1-hexene is an important commodity chemical that is used extensively on a large scale in the manufacture of linear low density and high density polyethylene (LLDPE and HDPE, respectively), it is becoming imperative that new routes that optimise selectivity for 1-hexene over less useful LAO product fractions are found [2]. Therefore, selective ethylene trimerisation has become the focus of much research in both academia and industry [3]. For example, Union Carbide [4–6], Chevron-Phillips [7–9] and BP [10, 11] have each developed their own homogeneous chromium-based catalyst packages for upgrading ethylene to 1-hexene. Each of these systems typically comprise a soluble chromium source, a ligand (often a tight bite angle diphosphine), and an alkyl aluminium activator such as methyl aluminoxane (MAO).

**Scheme 1:**
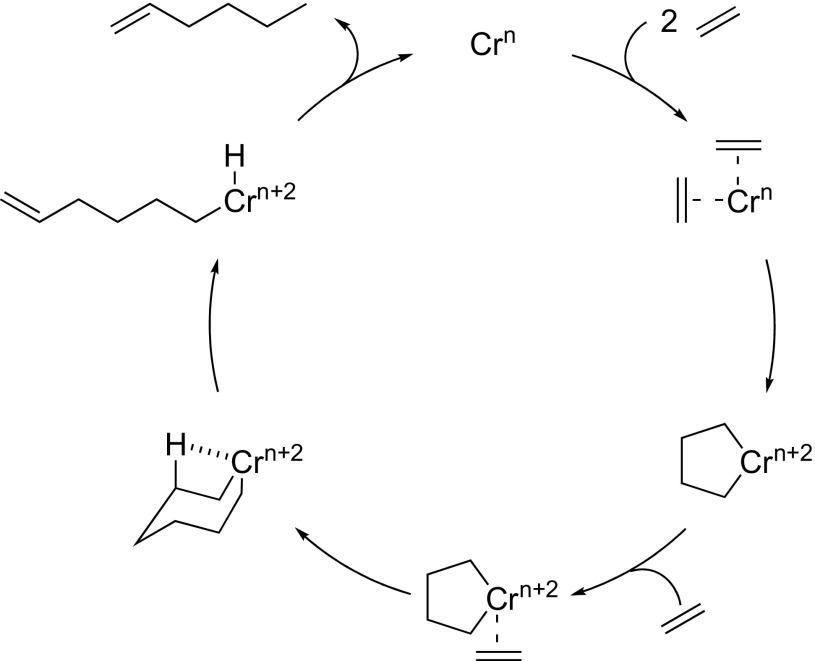
Ethylene trimerisation *via* a metallacycle mechanism

Conversely, there are relatively few examples of selective heterogeneous ethylene oligomerisation initiators having been reported [12–18]. Such heterogeneous systems could provide several advantages over their soluble counterparts in an industrial context, which include more efficient separation of the liquid product stream from the solid catalyst, the potential for a “solvent-free” continuous flow process, and minimisation of reactor fouling [19]. However, one barrier to the development of such heterogeneous systems is their complexity. Even for established homogeneous chromium-based selective olefin oligomerisation systems, where aspects of the general catalytic mechanism have been elucidated [1, 20], most notably the role of a metallacyclic reaction pathway (Scheme 1) [1, 21], there remains considerable debate about their precise mode of operation, including the formal oxidation states of the catalytically-active chromium species and specific aspects of ligand control [22–25]. Indeed, it is well-documented that catalytic performance relies on a complex interplay of factors including not only the structure of the molecular precursor and its supporting ligands, but also the nature of the aluminium activator, reaction solvent, and process conditions [26].

The origin of the selectivity towards ethylene trimerisation achievable with the established homogeneous systems is widely believed to result from operation of a metallacycle-based mechanism, as initially proposed by Manyik and later modified by Briggs (Scheme 1) [4, 6]. Here, chromium is thought to facilitate the oxidative coupling of ethylene, resulting in the formation of a five-membered chromacyclopentane [4]. Subsequent ethylene coordination and migratory insertion leads to the formation of a seven-membered metallacycle, which undergoes sequential β-hydride and reductive elimination steps to produce the target 1-hexene [6]. The selectivity of the process is thus controlled by the relative stabilities of the five- versus the seven-membered metallacycles, and by the rate of elimination of 1-hexene from the metallacycloheptane being faster than further insertion of ethylene to yield larger metallacycles [6].

This paper reports our findings from a fundamental study of the factors that influence the performance of a heterogeneous olefin trimerisation catalyst through use of a modification of a previously reported oxide-supported chromium initiator system developed by Monoi and Sasaki [18]. In particular, an assessment is made here of the relationship between the nature of the oxide support and its pre-treatment, the nature of the alkyl aluminium-based activator, and the reaction diluent against the performance of the heterogeneous ethylene trimerisation catalyst.

## Results and Discussion

The catalyst described by Monoi and Sasaki comprises an oxide-supported chromium pro-initiator, prepared through reaction of a well-defined molecular chromium(III) *tris*-(amide) complex with a partially dehydroxylated silica support, which is then activated using an alkyl aluminium reagent [18]. This system provides a convenient starting point here for developing future understanding.

### Preparation and Optimisation of Cr{N(SiMe_3_)_2_}_x_/Oxide_−600_ Ethylene Oligomerisation Initiators

#### Effect of Oxide Support

Partially-dehydroxylated SiO_2_ (Evonik Aeroperl 300/30), mixed SiO_2_–Al_2_O_3_ (Sigma Aldrich SiO_2_–Al_2_O_3_ Grade 135 catalyst support), and γ-Al_2_O_3_ (Alfa Aesar γ-Al_2_O_3_) were screened as potential catalyst supports in chromium-mediated ethylene oligomerisation. To enable comparison with the prior work of Monoi [18], each of the oxide materials was initially calcined at 600 °C for 24 h under a flow of dry nitrogen (the resulting materials being denoted as oxide_−600_). Subsequently, without exposure to the atmosphere, each of the oxide_−600_ materials was treated with a heptane solution of Cr{N(SiMe_3_)_2_}_3_ at room temperature (10 h) to afford materials with 0.71 wt% Cr loadings. The ethylene oligomerisation performance of the three oxide_−600_-bound chromium systems was then assessed in the slurry phase using modified methyl aluminoxane-12, MMAO-12, (Al:Cr = 15:1; toluene solution) as activator under identical test conditions, namely 8 barg ethylene, 120 °C, heptane solvent. For comparison, a homogeneous solution of Cr{N(SiMe_3_)_2_}_3_ was activated with MMAO-12 and tested in an analogous fashion.

The preliminary test results (Table 1) show that both the SiO_2−600_- and SiO_2_–Al_2_O_3−600_-supported systems afford hexenes as the principle products, with moderate selectivity to 1-hexene in both cases (Entries 2, 3), broadly in agreement with the previous observations of Monoi using a related silica-supported initiator [18]. In contrast, the γ-Al_2_O_3−600_-based system shows a complete switch in product selectivity not only favouring the formation of polyethylene (PE) rather than oligomerisation, but also showing significantly lower catalytic activity (Entry 4). Both the SiO_2_- and SiO_2_–Al_2_O_3_-supported systems demonstrate selectivity to C_6_ and C_10_ products rather than statistical product distributions. Catalytic tests undertaken using the soluble Cr{N(SiMe_3_)_2_}_3_ complex in combination with MMAO-12 as activator (Entry 1) give rise to extremely low productivity as well as a preference towards polymer formation. Together these observations are consistent with the oxide support playing an intimate role in the stabilisation of the active chromium species, as well as determining the nature of the catalytically-active chromium functionalities.

**Table 1:** Catalytic ethylene oligomerisation initiated by Cr{N(SiMe_3_)_2_}_3_, Cr{N(SiMe_3_)_2_}_x_/support (support = SiO_2 − 600_, SiO_2_-Al_2_O_3 − 600_, or γ-Al_2_O_3 − 600_) with MMAO-12 as activator

Entry	Support	C_4_ = ^*a*^ {wt%}	C_6_ = ^a^ (%1−C_6_=) {wt%}	C_8_ = ^a^ {wt%}	C_10_ = ^a^ {wt%}	C_12+_= ^a^ {wt%}	PE ^b^ {wt%}	Total activity {g g_Cr_^−1^ h^−1^}
1	No support	12	26 (81)	6	7	9	41	80
2	SiO_2−600_	1	61 (79)	2	16	6	13	2403
3	SiO_2_–Al_2_O_3−600_	1	71 (94)	3	10	3	12	1401
4	γ-Al_2_O_3−600_	2	3 (71)	3	3	4	85	237

*Reaction conditions* 27 μmol Cr (mass of oxide-supported catalyst = 0.2 g); 410 μmol MMAO-12 (Al:Cr 15:1); 60 ml heptane (solvent); 120 °C; stirrer speed 500 rpm; 8 barg ethylene pressure; nonane standard (1 ml); reaction time 0.5 h

^a^Determined by GC-FID relative to the internal standard nonane

^b^Polymer isolated by filtration, dried to constant mass and weighed

#### Effect of Aluminium Activator

It is well established that the nature of the Lewis acidic aluminium activator has a profound impact on the performance of early transition metal olefin oligomerisation systems, both in terms of activity as well as product selectivity [26, 27]. Although the identity of the active species responsible for selective homogeneously-catalysed ethylene trimerisation remains elusive, Bercaw et al. have provided compelling evidence that suggests that the Lewis acidic co-catalyst abstracts a ligand to produce a cationic Cr^III^ species, which then undergoes reductive elimination to form the active Cr^I^ trimerisation catalyst [28]. Consequently, it was important to explore whether such activator dependence was also observed for oxide-supported chromium amide-derived systems. To this end, a series of Lewis acidic co-catalysts, ^*i*^Bu_3_Al, isobutyl aluminoxane (IBAO), Me_3_Al, methyl aluminoxane (MAO), modified methyl aluminoxane-12 (MMAO-12) and Et_2_AlCl, were screened in combination with the Cr{N(SiMe_3_)_2_}_x_/SiO_2−600_ pro-initiator (Table 2).

**Table 2:** Effect of aluminium activator on ethylene oligomerisation performance using Cr{N(SiMe_3_)_2_}_x_/SiO_2 − 600_

Entry	Activator	C_4_ = ^a^ {wt%}	C_6_ = ^a^ (%1−C_6_=) {wt%}	C_8_ = ^a^ {wt%}	C_10_ = ^a^ {wt%}	C_12+_= ^a^ {wt%}	PE ^b^ {wt%}	Total activity {g g_Cr_^−1^ h^−1^}
1	No activator	0	0 (0)	0	0	0	0	0
2	MMAO-12	1	61 (79)	2	16	6	13	2403
3	^*i*^Bu_3_Al	3	33 (29)	0	0	7	56	1125
4	IBAO	12	19 (41)	0	1	62	6	358
5	Me_3_Al	5	44 (68)	4	3	9	35	243
6	MAO	0	9 (52)	0	0	14	76	969
7	Et_2_AlCl	2	4 (89)	3	2	16	74	114

*Reaction conditions* 27 μmol Cr (mass of Cr{N(SiMe_3_)_2_}_x_/SiO_2−600_ catalyst = 0.2 g); 410 μmol co-catalyst (Al:Cr 15:1); 60 ml heptane (solvent); 120 °C; stirrer speed 500 rpm; 8 barg ethylene pressure; nonane standard (1 ml); reaction time 0.5 h

^a^Determined by GC-FID relative to the internal standard nonane

^b^Polymer isolated by filtration, dried to constant mass and weighed

In line with the established trends demonstrated by homogeneous chromium-mediated ethylene oligomerisation initiators, the performance of the Cr{N(SiMe_3_)_2_}_x_/SiO_2−600_ system also exhibits a dependency on the nature of the Lewis acidic co-catalyst. Under the reaction conditions employed, MMAO-12 proved to be the optimal activator, both in terms of activity and selectivity towards 1-hexene (Table 2, Entry 2). Notably, in our hands, activation using IBAO afforded a system that was an order of magnitude less active and produced comparatively high levels of heavier oligomers (C_12+_) compared to the results described by Monoi [18]. While the origins of the enhanced performance of MMAO-12 in this initiator system remain obscure, it is likely that the greater thermal stability and better solubility of MMAO-12 in heptane compared with that of the other aluminium reagents, including MAO (Table 2, Entry 6), is a significant factor under the catalyst test conditions employed herein (i.e. heptane diluent, 120 °C) [29, 30]. However, the precise roles and modes of action of alkyl aluminium-based activators in both olefin oligomerisation and polymerisation are complex and generally remain rather poorly understood [30]. Consequently, it is possible that other factors may also contribute to differences observed between the performance of the various co-catalysts screened herein. These include potential coordination of alkyl aluminium species to the active chromium centre either directly or through ligation of the pendant amide groups, processes that can impede olefin coordination and provide a pathway for alkyl chain transfer [31–34]. Furthermore, since calcined oxides such as silica and alumina are established supports for alkyl aluminium activators (e.g. MAO and MMAO) themselves in both olefin oligomerisation and polymerisation catalysis, binding of the aluminium-based co-catalysts to the SiO_2−600_ cannot be ruled out, something that may also lead to a modification of the aluminoxanes through sequestration of residual trialkyl aluminium species [35, 36]. Studies to further probe the specific activator dependence observed here are on-going.

#### Effect of Diluent

Previous studies have shown that homogeneous ethylene oligomerisation processes are subject to substantial solvent effects [29, 37]. Accordingly, a series of batch ethylene oligomerisation runs using the heterogeneous Cr{N(SiMe_3_)_2_}_x_/SiO_2−600_ system were conducted to explore the impact of the organic diluent phase on catalytic performance (Table 3). The results indicate that the oxide-supported initiators perform best in aliphatic, non-polar solvents such as methylcyclohexane and heptane (Entries 1, 2). Conversely, use of aromatic solvents (i.e. Entries 3, 4) leads to a considerable drop in activity and an associated switch in product selectivity from oligomerisation towards PE formation. It has been reported previously that treatment of Cr^III^ complexes with alkyl aluminium reagents in aromatic solvents, for example Cr(acac)_3_/AlMe_3_ in toluene [38], leads to the formation of reduced chromium(I) sandwich complexes of the type [Cr(η^6^-arene)_2_]^+^ [22], something that has been ascribed to account for the deactivation of homogeneous chromium oligomerisation systems in such solvents [29, 37]. Hence, it is likely that analogous Cr^I^ arene species are also formed during the activation of the oxide-bound chromium amide species, something that is consistent with the observed drop in activity.

**Table 3:** Diluent effects on ethylene oligomerisation performance using Cr{N(SiMe_3_)_2_}_x_/SiO_2−600_

Entry	Diluent	C_4_ = ^*a*^ {wt%}	C_6_ = ^a^ (%1−C_6_=) {wt%}	C_8_ = ^a^ {wt%}	C_10_ = ^a^ {wt%}	C_12+_= ^a^ {wt%}	PE ^b^ {wt%}	Total activity {g g_Cr_^−1^ h^−1^}
1	Heptane	1	61 (79)	2	16	6	13	2403
2	Methylcyclohexane	1	61 (72)	4	12	6	16	2132
3	Chlorobenzene	1	1 (85)	1	1	2	94	792
4	Toluene	2	51 (96)	3	3	5	36	449

*Reaction conditions* 27 μmol Cr (mass of Cr{N(SiMe_3_)_2_}_x_/SiO_2−600_ catalyst = 0.2 g); 410 μmol MMAO-12 co-catalyst (Al:Cr 15:1); 60 ml solvent; 120 °C; stirrer speed 500 rpm; 8 barg ethylene pressure; nonane standard (1 ml); reaction time 0.5 h

^a^Determined by GC-FID relative to the internal standard nonane

^b^Polymer isolated by filtration, dried to constant mass and weighed

### Understanding the Nature and Catalytic Behaviour of Cr{N(SiMe_3_)_2_}_x_/SiO_2−600_

#### Raman Spectroscopic Analysis

**Fig. 1:**
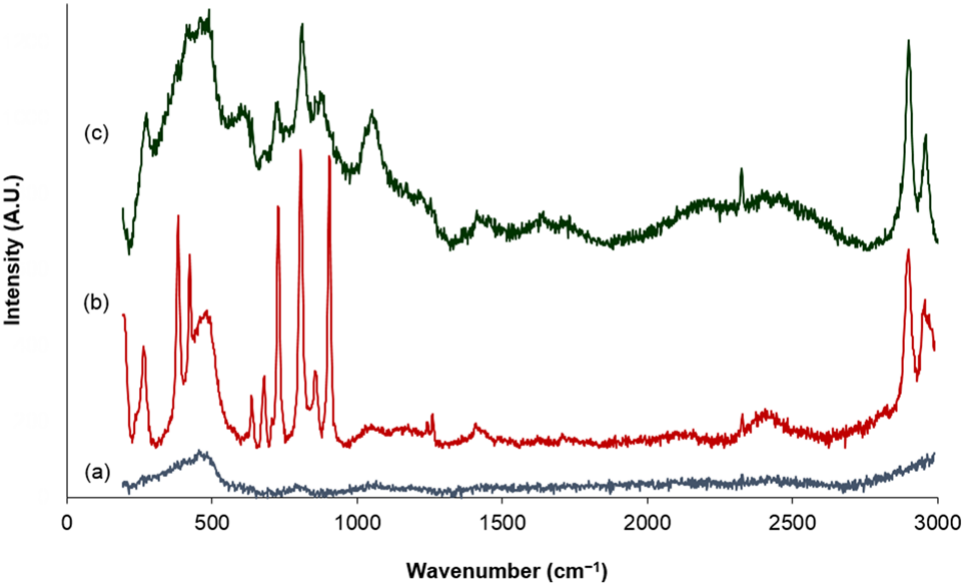
Solid-state Raman spectra of (**a**) SiO_2−600_, (**b**) Cr{N(SiMe_3_)_2_}_3_, and (**c**) Cr{N(SiMe_3_)_2_}_x_/SiO_2−600_

In order to understand the mode of action of this class of heterogeneous chromium-based olefin oligomerisation initiator, it is essential to develop insight into the nature of the surface-bound metal species. It is assumed that the Cr{N(SiMe_3_)_2_}_3_ precursor will react at the surface of SiO_2−600_ *via* the residual silanol sites eliminating the corresponding amine, HN(SiMe_3_)_2_. This type of reaction pathway is indeed supported by the Raman spectroscopic analysis of the resulting Cr{N(SiMe_3_)_2_}_x_/SiO_2−600_ material, which exhibits bands consistent with the presence of covalent Cr–O–Si linkages (1050 cm^−1^), and with the retention of amide ligands (Fig. 1) [39, 40].

#### Solid-State ^29^Si NMR Spectroscopic Analysis of Cr{N(SiMe_3_)_2_}_x_/SiO_2−600_

Although it was previously envisaged that for chromium-based systems, the operation of a metallacycle-based ethylene trimerisation reaction mechanism precluded the formation of longer chain oligo-/poly-meric products [4, 6, 41], more recent studies have since demonstrated that long-chain oligomers may also originate from a metallacyclic reaction manifold [42, 43]. In our work, however, the selective production of 1-hexene is not only accompanied by the formation of decenes, something that is likely to arise from secondary metallacycle-based ethylene/1-hexene co-trimerisation processes [43–45], but also polyethylene. These findings suggest that more than one catalytically active chromium species may be present at the surface of the partially dehydroxylated SiO_2−600_ catalyst support. Therefore, to investigate this possibility, the intrinsic paramagnetic nature of the oxide-immobilised chromium species has been exploited to help probe indirectly the nature of the supported transition metal species using solid-state ^29^Si NMR spectroscopy. This builds upon a previous study that demonstrated that magic-angle spinning (MAS) ^29^Si NMR spectroscopy provides information regarding the nature of the catalytically-relevant paramagnetic chromium species present in the related long-standing commercial “chromium” on silica Phillips ethylene polymerisation catalyst [46]. These experiments exploit the fact that the time constant for dipolar coupling relaxation increases with distance as *r*^*6*^, so the recovered magnetisation at time *t* after saturation will be that of the spins contained in a sphere of radius *r* corresponding to *t*^*1*/*6*^ [47]. Since dipolar coupling is typically transmitted over long distances by nuclear spin–spin diffusion, magic-angle spinning has the affect of averaging the secular component of the dipolar coupling between nuclear spins, which effectively quenches nuclear-spin diffusion. Together, this provides a cut-off above which nuclear-spin relaxation is observed, hence providing a measure of proximity between the paramagnetic chromium metal centre and ^29^Si nuclei [48].

**Fig. 2:**
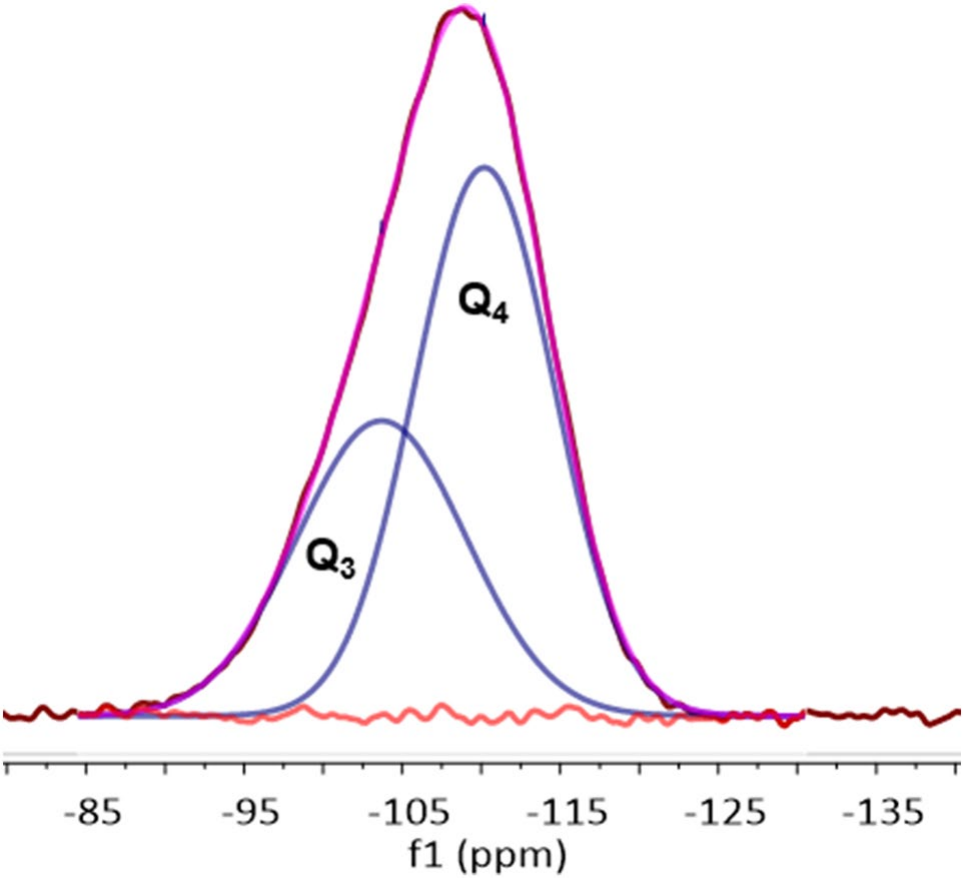
Deconvoluted solid-state ^29^Si DE MAS NMR spectrum of Cr{N(SiMe_3_)_2_}_x_/SiO_2−600;_ 79 MHz, rotation 8 KHz

**Scheme 2:**
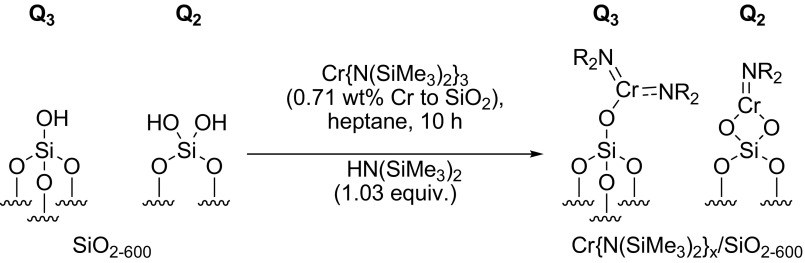
Immobilisation of Cr{N(SiMe_3_)_2_}_3_ on SiO_2−600_

To this end, a sample of the Cr{N(SiMe_3_)_2_}_x_/SiO_2−600_ pro-initiator was packed into an airtight rotor inside a nitrogen-filled glove box, and sealed under an inert atmosphere, prior to solid-state ^29^Si direct excitation (DE) MAS NMR spectroscopic analysis (Fig. 2). Compared to the longitudinal relaxation rates (*T*_*1*_^−1^) of ^29^Si nuclei present in SiO_2−600_ (*T*_*1*_^−1^ = 15.9 × 10^−3^ s^−1^) the presence of a paramagnetic Cr^III^ species increased the *T*_*1*_^−1^ of the corresponding nuclei in Cr{N(SiMe_3_)_2_}_x_/SiO_2−600_ (*T*_*1*_^−1^ = 46.5 × 10^−3^ s^−1^), in addition to a fast-relaxing component (4.4 s^−1^). The latter results in line broadening of the resonances associated with the Q_3_ and Q_2_ environments (see Scheme 2), which were previously found to be in a 29:1 ratio by deconvolution (Gaussian distribution function) of the ^29^Si NMR spectrum of SiO_2−600_. Most notably, the resonance associated with the Q_2_ environments in Cr{N(SiMe_3_)_2_}_x_/SiO_2−600_ is broadened to such an extent that it is proposed to be lost in the baseline. Since *T*_*1*_ is directly proportional to the distance between the unpaired electron and the nucleus being observed by NMR spectroscopy (to the power of six) [49], it may be inferred that the ^29^Si nuclei corresponding to Q_2_ and Q_3_ sites are in close proximity to the supported chromium(III) species. In addition, the chromium(III) complex acts as a paramagnetic NMR shift reagent, such that the signal associated with the Q_3_^29^Si nuclei is shifted to a lower frequency (Δδ = − 5 ppm) than that for the Q_4_ environment (Δδ = − 1 ppm). Taking these two observations together with the fact that the change in resonant frequency brought about by a through-space dipolar interaction is inversely proportional to the distance between the unpaired electron and the nucleus being observed by NMR spectroscopy (to the power of three) [50], we propose that Cr{N(SiMe_3_)_2_}_3_ has reacted with both Q_2_ and Q_3_ silanols at the surface of SiO_2600_ to give two different types of silica-bound species.

#### Cr{N(SiMe_3_)_2_}_x_/SiO_2−600_ Titration Experiment

To further confirm the presence of two different surface-bound chromium species, a titration experiment was conducted in which a sample of SiO_2−600_ (3.15 mmol_OH_ g^−1^) was treated with a heptane solution of Cr{N(SiMe_3_)_2_}_3_ to a chromium loading of 0.71 wt%. This resulted in the evolution of HN(SiMe_3_)_2_ (1.03 molar equivalents) as a result of the reaction with the surface silanols. Since the ratio of Q_2_–Q_3_ silanol sites in SiO_2−600_ was determined to be 1:29, and the chromium metal loading of the resulting Cr{N(SiMe_3_)_2_}_x_/SiO_2_ pro-initiator was verified to be 0.71 wt% by ICP-OES analysis, it is proposed that Cr{N(SiMe_3_)_2_}_3_ reacts with both Q_2_ and Q_3_ surface silanol sites resulting in the formation of one or two Cr–O bonds, respectively, as demonstrated by evolution of 1.03 equivalents of amine (Scheme 2).

#### Impact of Support Calcination Temperature

Since there are at least two different types of supported chromium(III) species at the surface of Cr{N(SiMe_3_)_2_}_x_/SiO_2−600_, which result from the presence of both Q_2_ and Q_3_ sites in a ratio of 1:29 for SiO_2−600_, a second catalytic system was prepared using silica calcined at 200 rather than 600 °C, in order to increase the relative concentration of Q_2_ silanols with respect to Q_3_. The effects of calcination of the SiO_2_ support material were explored by thermogravimetric analysis (TGA) and solid-state ^29^Si NMR spectroscopy. The latter technique provides direct quantitative information on the relative changes in silanol content and nature as a function of temperature.

**Fig. 3:**
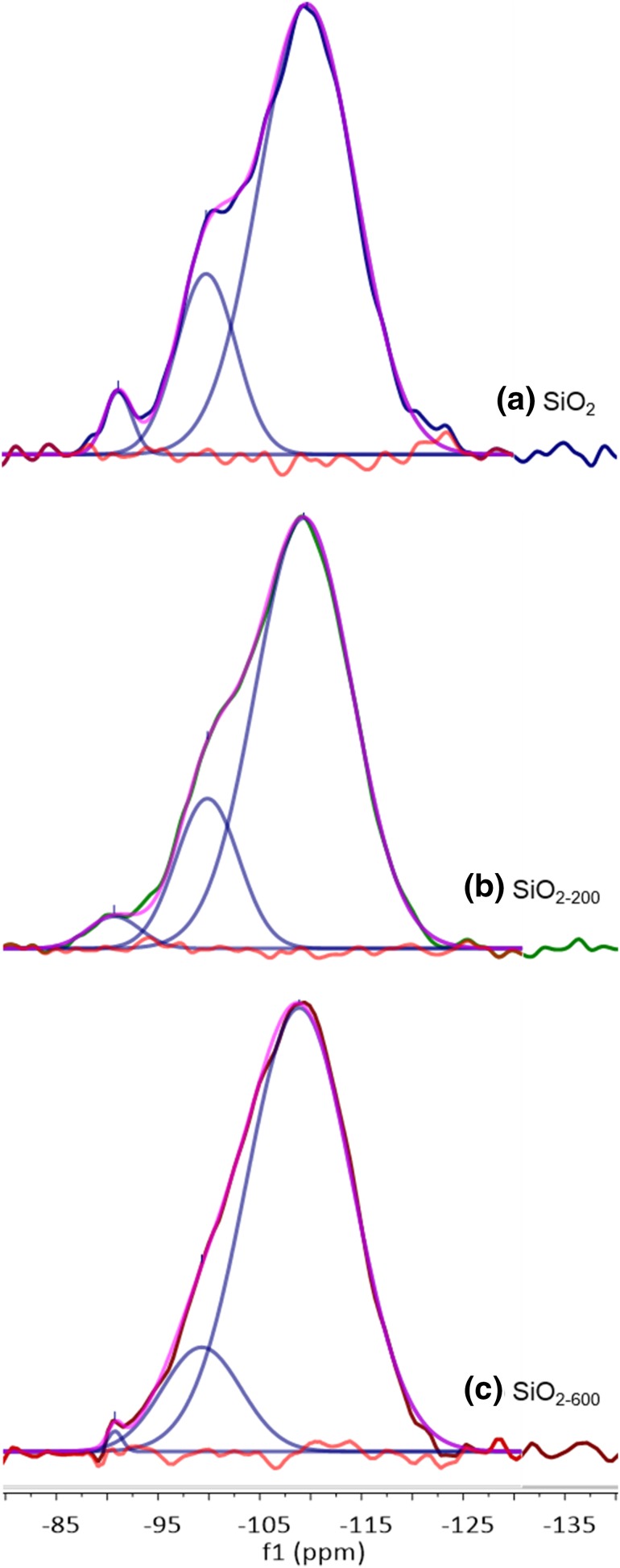
Deconvoluted (Gaussian distribution) solid-state ^29^Si DE NMR spectra (79 MHz, rotation 6 KHz): (**a**) untreated SiO_2_; (**b**) SiO_2−200_, (**c**) SiO_2−600_

Solid-state ^29^Si DE MAS NMR spectroscopy was used to quantify the change in the relative proportions of the Q_2_ (− 91 ppm), Q_3_ (− 99 ppm) and Q_4_ (− 110 ppm) sites of the Aeroperl 300/30 silica as a function of calcination temperature (Fig. 3; Table 4), with spectral assignments made in accordance with a previous NMR spectroscopic study [51]. The deconvoluted ^29^Si NMR spectra demonstrate that as the temperature of calcination is raised, the concentration of Q_2_, Q_3_ and vicinal silanols is attenuated. Nevertheless, although both the SiO_2−200_ and SiO_2−600_ materials exhibit three resonances in their ^29^Si NMR spectra, it is impossible to discriminate between vicinal and Q_3_ silanols as their characteristic resonance frequencies overlap at ~ − 100 ppm [51]. However, since it is well-established that vicinal silanols condense at calcination temperatures of 400 °C and above [52, 53], it is proposed that the sample calcined at 200 °C (SiO_2−200_) retains some vicinal silanol functionalities, as well as both Q_2_ and Q_3_ silanol sites. Contrastingly, the SiO_2−600_ material revealed that both Q_2_ and Q_3_ sites are present.

**Table 4:** Relative proportions of Q_2_, vicinal, Q_3_ and Q_4_ sites present in untreated SiO_2_ (Aeroperl 300/30 silica), SiO_2−200_, and SiO_2−600_ assigned based on a Gaussian distribution curve fit of the corresponding ^29^Si DE NMR spectra (79 MHz, rotation 6 KHz)

Sample	Q_2_ silanol (− 91 ppm) (%) 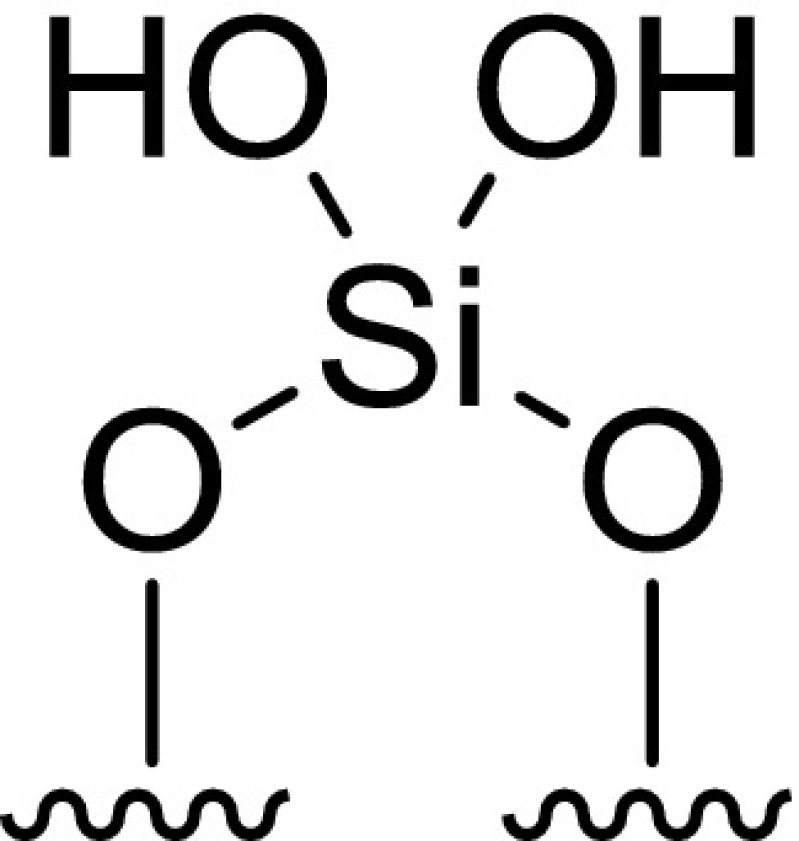	Vicinal and Q_3_ silanol (− 99 ppm) (%) 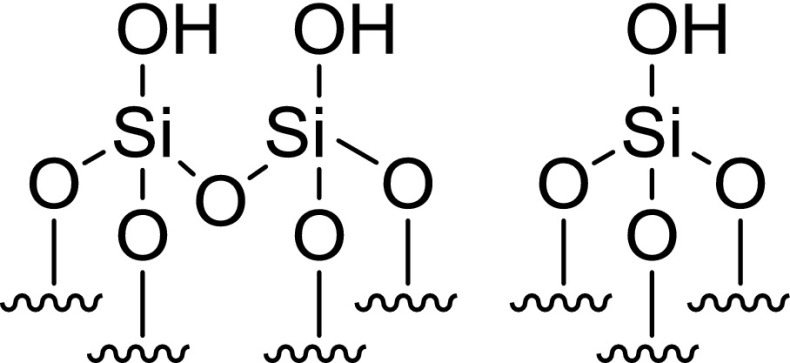	Q_4_ site (− 110 ppm) (%) 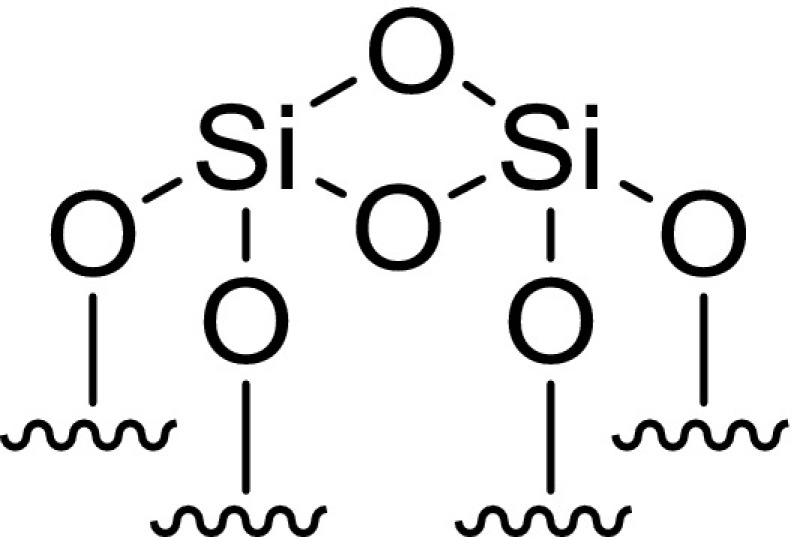
SiO_2_	3	20	77
SiO_2−200_	3	18	79
SiO_2−600_	< 1	15	85

**Fig. 4:**
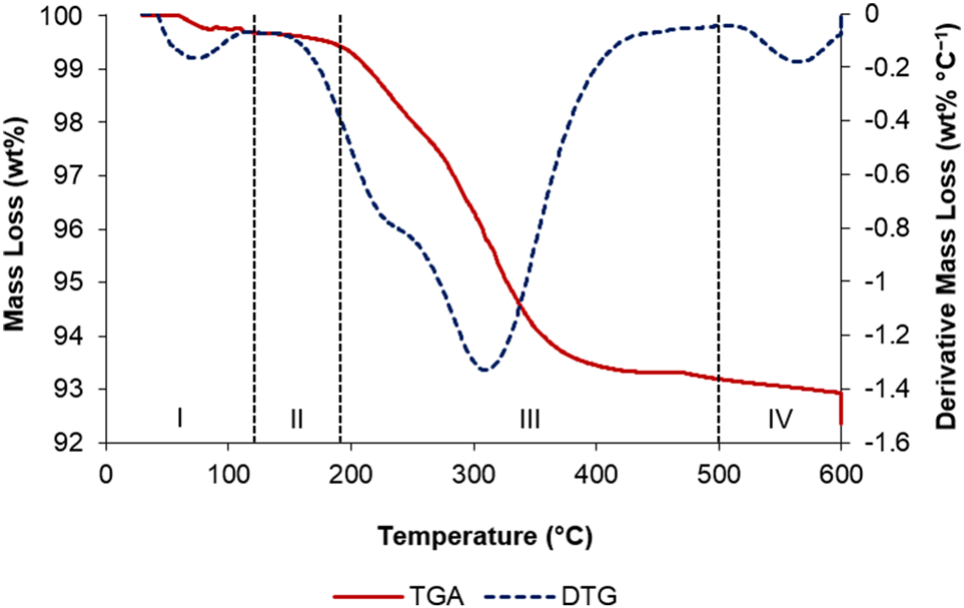
TGA/DTG profiles for Aeroperl 300/30 SiO_2_; heating rate 30 °C min^− 1^ to 600 °C; assignment of profile regions I–IV given in the text

In order to further differentiate the nature of the reactive surface silanol species present on the silica surfaces following calcination, a TGA study was undertaken in parallel. As expected from previous reports concerning a number of different silicas [53], the calcination of Aeroperl 300/30 occurs over four distinct temperature regimes, as evidenced by TGA/DTG (Fig. 4, I–IV): loss of physisorbed water (dehydration) between 50 and 120 °C (I) and 120–190 °C (II); condensation of vicinal, isolated (Q_3_) and geminal (Q_2_) silanols (i.e. dehydroxylation) between ~ 190 and 450 °C (III); and further dehydroxylation of Q_2_ and Q_3_ silanols above 500 °C (IV). Consequently, the silica sample calcined at 200 °C (SiO_2−200_) may be regarded as essentially dehydrated silica, retaining a significant concentration of Q_2_, Q_3_ and vicinal silanol functionalities in accordance with the solid-state NMR spectroscopic data (Table 4). In contrast, from combining the TGA and NMR spectroscopic studies, the surface of the SiO_2−600_ material is found to be both dehydrated and partially dehydroxylated, thus leaving it with primarily Q_3_ silanol groups together with Q_4_ sites and a low concentration of residual Q_2_ species (i.e. Q_3_:Q_2_ = 29:1). These differences in the nature and hence reactivity of the surfaces of SiO_2−200_ and SiO_2−600_ will directly lead to the generation of different chromium species following reaction of each of these materials under anhydrous conditions with Cr{N(SiMe_3_)_2_}_3_.

Subsequently, SiO_2−200_ was treated with Cr{N(SiMe_3_)_2_}_3_ using an analogous procedure to that used for the preparation of Cr{N(SiMe_3_)_2_}_x_/SiO_2−600_, and the catalytic performance of the resulting material evaluated in combination with MMAO-12 under standard test conditions (Table 5). Not only is the resulting Cr{N(SiMe_3_)_2_}_x_/SiO_2−200_ system a much less active initiator (Entry 1), but it also shows a dramatic switch in selectivity towards the formation of PE compared with the system prepared using SiO_2−600_ (Entry 2). This is consistent with previous preliminary observations made by Monoi and co-workers [18, 54].

**Table 5:** Impact of silica pre-calcination temperature on catalytic ethylene oligomerisation performance of Cr{N(SiMe_3_)_2_}_x_/oxide (oxide = SiO_2−200_ or SiO_2−600_)

Entry	Catalyst support	C_4_ = ^a^ {wt%}	C_6_ = ^a^ (%1−C_6_=) {wt%}	C_8_ = ^a^ {wt%}	C_10_ = ^a^ {wt%}	C_12+_= ^a^ {wt%}	PE ^b^ {wt%}	Total activity {g g_Cr_^−1^ h^−1^}
1	SiO_2−200_	1	19 (93)	2	2	4	72	1363
2	SiO_2−600_	1	61 (79)	2	16	6	13	2403

*Reaction conditions* 27 μmol Cr (mass of oxide-supported catalyst = 0.2 g); 410 μmol co-catalyst (Al:Cr 15:1); 60 ml heptane (solvent); 120 °C; stirrer speed 500 rpm; 8 barg ethylene pressure; nonane standard (1 ml); reaction time 0.5 h

^a^Determined by GC-FID relative to the internal standard nonane

^b^Polymer isolated by filtration, dried to constant mass and weighed

**Scheme 3:**
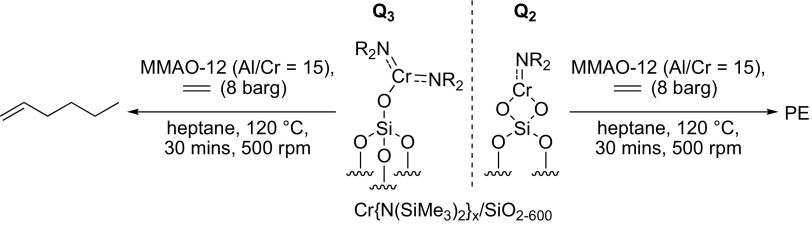
Proposed Cr-based pro-initiators responsible for ethylene trimerisation and PE formation

Since the ratio of Q_2_:Q_3_ silanols increases at higher support calcination temperatures, it is proposed that chromium amide species bound to Q_2_ sites favour PE formation (through a classical Cossee-Arlman chain growth mechanism [55–57]), whereas Q_3_-bound chromium species mediate ethylene trimerisation *via* a supported variant of the metallacycle mechanism (Scheme 1) [6], as shown in Scheme 3. Parallels may therefore be drawn between the *heterogeneous* Cr{N(SiMe_3_)_2_}_x_/SiO_2−600_ pro-initiator described herein, the more well-established *homogeneous* Cr-based ethylene trimerisation systems [1, 3], and indeed the supported “Phillips” Cr/SiO_2_ polymerisation catalyst [58, 59].

## Conclusions

Preliminary catalyst screening of Cr{N(SiMe_3_)_2_}_x_/oxide_−600_-based oligomerisation pro-initiators demonstrated that catalytic performance is intimately linked to the nature of the oxide support, aluminium activator, and organic diluent. In our hands the best performing ethylene trimerisation initiator comprise Cr{N(SiMe_3_)_2_}_x_/SiO_2−600_ and MMAO-12 operated as a slurry in heptane. Based on a combined TGA and solid-state ^29^Si NMR spectroscopic study, two distinct silica-immobilised chromium species are present at the surface of Cr{N(SiMe_3_)_2_}_x_/SiO_2−600_. It is proposed that in combination with the aluminium activator MMAO-12, =SiO_2_CrN(SiMe_3_)_2_ species resulting from reaction of Cr{N(SiMe_3_)_2_}_3_ with Q_2_ silanol sites, are responsible for polyethylene formation, while selective ethylene trimerisation is mediated by ≡SiOCr{N(SiMe_3_)_2_}_2_ derived from Q_3_ silanols.

## General Experimental

Unless stated otherwise, all manipulations were carried out under an atmosphere of dry nitrogen using standard Schlenk line techniques or in an Innovative Technologies nitrogen-filled glovebox. All glassware was oven-dried before use. Dry solvents were obtained from an Innovative Technologies SPS facility and degassed prior to use by three freeze-pump-thaw cycles, unless otherwise stated. Pentane, heptane, methylcyclohexane and nonane were dried over CaH_2_, distilled and degassed. Chlorobenzene was dried over P_2_O_5_, distilled and degassed. Evonik Aeroperl 300/30 SiO_2_ (described herein as SiO_2_), Sigma Aldrich SiO_2_–Al_2_O_3_ Grade 135 catalyst support (13 wt% Al; described herein as SiO_2_–Al_2_O_3_) [60], and Alfa Aesar γ-Al_2_O_3_ (1/8″ pellets ground and sieved to < 250 μm; described herein as γ-Al_2_O_3_) were used as catalyst supports. Ethylene (BOC) was passed through a moisture scrubbing column containing molecular sieves (Sigma Aldrich; 3A, 4A, and 13X) that had previously been activated at 400 °C for three hours under dynamic vacuum (i.e. 0.05 mbar), before being cooled to RT and stored under ethylene. All other chemicals, unless stated, were obtained from Sigma Aldrich or Alfa Aesar and used without further purification. Modified methyl aluminoxane-12 (MMAO-12); Sigma Aldrich, 7 wt% solution in toluene; approx. molecular formula: [(CH_3_)_0.95_(n-C_8_H_17_)_0.05_AlO]_n_.

Isobutyl aluminoxane (IBAO) was prepared according to a modification of a previously disclosed protocol [61]. Distilled, deionised water (20 mL) was degassed by purging with N_2_ at a rate of 2 mL s^−1^. An ampoule was charged with ^*i*^Bu_3_Al (25 wt% solution in toluene; 25 mL, 5.3 g, 0.0267 moles), which was then cooled in an ice bath to 4 °C. An aliquot (0.85 molar equivalents) of distilled, deionised and degassed H_2_O (0.41 mL, 0.0228 moles) was added cautiously drop-wise to the cool, stirring solution of ^*i*^Bu_3_Al. The reaction mixture was then allowed to warm to RT, and stirred for a further 10 h. The resulting colourless solution was stored at RT in an ampoule under N_2_ and used, as prepared, without further analysis.

The complex Cr{N(SiMe_3_)_2_}_3_ was synthesised according to the protocol previously reported by Bradley and isolated as a dark green air-/moisture-sensitive solid, which was handled under an inert atmosphere [62]. *Anal*. Calc. for C_18_H_54_N_3_CrSi_6_: C, 40.55; H 10.21; N 7.88 Found: C, 40.51; H, 10.30; N, 7.71%. IR (KBr, Nujol *ν*_max_/cm^−1^) 1263, 1254, 910, 860, 794, 760, 708, 678, and 619 (lit. [40], 1260, 1250, 902, 865, 840, 820, 790, 758, 708, 676 and 620). Raman (solid, 532 nm, *ν*_max_/cm^−1^) 2956, 2898, 1260, 1240, 904, 855, 805, 728, 707, 679, 636, 424 and 382.

Brunauer–Emmett–Teller (BET) specific surface area analysis (SSA) and Barrett–Joyner–Halenda (BJH) pore size and volume analyses were completed using a Micromeritics instrument. Thermogravimetric (TGA) analyses were completed using a Perkin Elmer Pyris 1 TGA, coupled to a Hiden HPR 20 MS unit purged with helium gas. Solid-state ^1^H (400 MHz frequency; 13 KHz rotation rate), ^27^Al (104 MHz; 13 KHz spinning rate) magic spinning angle (MAS) and ^29^Si (79 MHz; 6–8 KHz rotation rate) direct excitation (DE) nuclear magnetic resonance (NMR) spectroscopic analyses (Varian VNMRS) were completed with samples packed into an airtight rotor inside a nitrogen-filled glove box, and sealed under an inert atmosphere. Solid-state NMR samples were referenced to external Si(CH_3_)_4_ (^1^H, ^29^Si) or 1M Al(NO_3_)_3(aq)_ (^27^Al). The ^29^Si NMR resonances attributed to geminal (Q_2_), isolated (Q_3_) and bulk silica (Q_4_) were quantified using a Gaussian distribution curve fit using MestreNova (MestreLab). Spin–lattice relaxation times were measured using a saturation-recovery method. A five-parameter fit was used to model the result, including a two-component exponential recovery plus baseline. Raman spectroscopy was conducted using a Horiba LabRAM-HR spectrometer equipped with a 532 nm frequency-doubled Nd:YAG laser and a 1800 lines/mm grating. The samples were loaded into a standard glass J-Young NMR tube inside a nitrogen-filled glove box and sealed under an inert atmosphere.

Gas chromatographic (GC) analyses were performed on a Perkin Elmer Clarus 400 system equipped with a PONA (50 m × 0.20 mm × 0.50 μm) capillary column. Analytes were detected using a flame ionisation detector (FID). The oven temperature was maintained at 40 °C for 10 min, before the temperature was increased to 170 °C at a rate of 20 °C min^−1^. This temperature was maintained for 5 min. Subsequently, the column was heated further to 300 °C, again at a rate of 20 °C min^−1^. The temperature was maintained at 300 °C for 12 min before the system was allowed to cool to 40 °C. The GC-FID analysis total run time was 40 min.

## General Procedures for Calcination of Oxide Support Materials

Using a variation of a previously reported methodology [18], a quartz tube (20 mm I.D.) fitted with a porous quartz frit was sequentially charged with previously acid-washed quartz wool (H. M. Baumbach) and the oxide-based catalyst support (5.0 g) to form a solid plug. The quartz tube was then placed vertically inside a tube furnace, such that the oxide was centred in the furnace; a thermocouple was attached to the outside of the quartz tube and located level with the centre of the oxide bed. Oxygen-free nitrogen gas, previously dried by passage through a drying column consisting of CaCl_2_ and P_2_O_5_, was passed down through the oxide bed (1 mL s^−1^), exiting the system *via* a silicon oil bubbler. The oxide was heated to 600 °C (ramp rate = 10 °C min^−1^) and then maintained at 600 °C for 24 h under a flow of N_2_. Subsequently, the calcined material was allowed to cool to room temperature under a flow of N_2_, before being transferred under vacuum into a glovebox without exposure to air. Supports are classified by the temperature at which they were calcined, e.g. SiO_2−600_ denotes silica calcined at 600 °C for 24 h under a flow of N_2_. The SiO_2−200_ support material was prepared from SiO_2_ using an analogous protocol, but being held at only 200 °C for 24 h.

## General Analyses of Oxide Support Materials

### Specific Surface Area and Pore Volume/Size Analysis

The BET specific surface area (SSA) and BJH pore volume/size distributions for Evonik Aeroperl 300/30 fumed SiO_2_, Sigma Aldrich SiO_2_–Al_2_O_3_, and Alfa Aesar γ-Al_2_O_3_ catalyst supports have been determined (Table 6). A sample of each untreated oxide-based support was degassed at 140 °C with a nitrogen purge for 1 h, prior to BET specific surface area and isotherm measurements.

**Table 6:** Specific surface area, pore size/volume analyses, obtained using BET and BJH methods

Oxide support	SSA (m^2^ g^−1^)	Pore volume (cm^3^ g^−1^)	Pore diameter (Å)
SiO_2_	285	1.85	260
SiO_2_–Al_2_O_3_	506	0.75	59
γ-Al_2_O_3_	244	0.76	124

### Solid-State NMR Spectroscopic Analysis of Oxide Supports

Evonik Aeroperl 300/30 fumed SiO_2_, Sigma Aldrich SiO_2_–Al_2_O_3_ and Alfa Aesar γ-Al_2_O_3_ catalyst supports were analysed using solid-state ^1^H, ^27^Al and ^29^Si DE MAS NMR spectroscopy.

#### Evonik Aeroperl 300/30 Fumed SiO_2_

^1^H DE MAS NMR (400 MHz, solid, 13 KHz rotation, 1 s recycle, 160 repetitions) δ = 3.7. ^29^Si DE MAS NMR (79 MHz, solid, 6 KHz rotation, 120 s recycle, 547 repetitions) δ = − 91 (Q_2_), − 100 (Q_3_), − 110 (Q_4_).

#### Sigma Aldrich Grade 135 SiO_2_–Al_2_O_3_

^1^H DE MAS NMR (400 MHz, solid, 13 KHz rotation, 1 s recycle, 160 repetitions) δ = 7.0, 5.0. ^27^Al DE MAS NMR (104 MHz, solid, 13 KHz rotation, 0.2 s recycle, 750 repetitions) δ = 56 (AlO_4_), 4 (AlO_6_). ^29^Si DE MAS NMR (79 MHz, solid, 6 KHz rotation, 30 s recycle, 1824 repetitions) δ = − 91 (Q_2_), − 102 (Q_3_), − 110 (Q_4_).

#### Alfa Aesar γ-Al_2_O_3_ (Powder Sieved to < 250 μm)

^1^H DE MAS NMR (400 MHz, solid, 13 KHz rotation, 1 s recycle, 160 repetitions) δ = 5.0. ^27^Al DE MAS NMR (104 MHz, solid, 13 KHz rotation, 0.2 s recycle, 3950 repetitions) δ = 64 (AlO_4_), 5 (AlO_6_).

The thermogravimetric profile (TGA/DTG) of SiO_2_ was measured between 30 and 600 °C, at a rate of 30 °C min^1^, and then held at 600 °C for 24 h.

### Analysis of SiO_2−200_

^29^Si DE MAS NMR (79 MHz, solid, 6 KHz rotation, 120 s recycle, 500 repetitions) δ = − 91 (Q_2_), − 100 (Q_3_), − 109 (Q_4_).

### Analysis of SiO_2−600_

^1^H DE MAS NMR (400 MHz, solid, 6 KHz rotation, 5 s recycle, 32 repetitions) δ = 1.9. ^29^Si DE MAS NMR (79 MHz, solid, 6 KHz rotation, 120 s recycle, 500 repetitions) δ = − 91 (Q_2_), − 99 (Q_3_), − 109 (Q_4_). Raman (solid, 532 nm, *ν*_max_/cm^−1^): 455.

#### Estimation of Residual Silanol Concentration for SiO_2−600_ by Titration Para-Tolylmagnesium Bromide

A Schlenk was charged with SiO_2−600_ (0.2116 g) inside a glovebox and sealed under N_2_. The calcined material was suspended in heptane (10 mL), stirred at 200 rpm *via* a Teflon-coated magnetic stirrer bar and then cooled to 5 °C using an ice/water bath, prior to being reacted with a Et_2_O solution of *para*-tolylmagnesium bromide (1.8 mL, 2 M, 3.6 mmol), which was added slowly *via* a syringe. The stirred suspension was then allowed to warm to RT. After 1 h, the reaction was cooled to 0 °C using an ice/water bath and quenched with propanal (5 mL, 69.7 mmol), before nonane (1.0 mL, 5.6 mmol) was added as an internal standard. An aliquot of the organic phase was filtered through a plug of cotton wool/Celite and subsequently analysed by GC-FID. The concentration of residual silanols was determined, from the quantity of liberated toluene, to be 3.15 mmol_OH_ g^−1^.

#### Quantification of Amine Liberated Through Reaction of SiO_2−600_ with Cr{N(SiMe_3_)_2_}_3_

An ampoule was charged with freshly calcined SiO_2−600_ (2.89 g, 0.048 moles) inside a glovebox and sealed under N_2_. The ampoule was connected to a Schlenk line *via* a vacuum transfer side arm. A stock solution of Cr{N(SiMe_3_)_2_}_3_ in heptane (50.5 mL, 0.0078 M, 0. 39 mmol; Cr/SiO_2_ = 0.71 wt%) was added portion-wise to the reaction vessel using a dry, degassed syringe. The resulting white solid, suspended in a green solution, was stirred for 10 h at RT, by which time the solution had become colourless and the solid green. ICP-OES analysis confirmed no residual chromium in the organic phase. The combined reaction mixture was frozen at − 196 °C and the reaction vessel evacuated (0.1 mbar). Upon thawing, all volatile components were isolated by vacuum transfer to afford a colourless organic solution. Subsequently nonane (1.0 mL 5.6 mmol) was added to this solution, before an aliquot of the resulting mixture was collected, passed through a plug of cotton wool/Celite, and analysed by GC-FID to quantify the amount of HN(SiMe_3_)_2_ liberated on reaction of Cr{N(SiMe_3_)_2_}_3_ with a known quantity of SiO_2−600_. The mole ratio of Cr : HN(SiMe_3_)_2_ was determined to be 1:1.03.

### General Protocol for the Preparation of the Oxide-Supported Chromium Catalysts

A Schlenk was charged with the partially dehydroxylated oxide support (2.0 g) inside a glove box and sealed under N_2_. The Schlenk was connected to a vacuum line, evacuated and re-filled with dry N_2_ three times, then charged with a stock solution of Cr{N(SiMe_3_)_2_}_3_ in heptane (0.0078 M, 35 mL, 0.27 mmol; 0.71 wt% Cr). The reaction mixture was stirred at 500 rpm *via* a Teflon-coated magnetic stirrer bar for 10 h at RT. At the end of this period, the solution had changed from green to colourless, while the solid had turned green. All volatile components were then removed *in vacuo* and the resulting green solid transferred into a nitrogen-filled glove box and stored at ambient temperature. The extent of Cr uptake was assessed *via* ICP-OES analysis of the impregnated oxide materials.

### General Protocol for the Determination of Chromium Loading of Cr{N(SiMe_3_)_2_}_x_/Oxide by ICP-OES

A known mass of the Cr{N(SiMe_3_)_2_}_x_/oxide (0.0063 g; 0.71 wt% Cr) was charged into a polypropylene vial under air, and suspended in an aqueous solution of HCl (1.5 mL, 37% w/w, 12.7 mmol). Following 10 h standing at RT the mixture was carefully diluted with deionised water (13.5 mL), prior to ICP-OES analysis. The ICP-OES instrument was calibrated using standard aqueous solutions of Cr(NO_3_)_3_·6H_2_O.

### Analysis of Cr{N(SiMe_3_)_2_}_x_/SiO_2−600_

The ethylene trimerisation pro-initiator was analysed using both Raman and solid-state ^29^Si NMR spectroscopies.

^1^H DE MAS NMR (400 MHz, solid, 6 KHz rotation, 5 s recycle, 32 repetitions) δ = 0.15. ^29^Si DE MAS NMR (79 MHz, solid, 8 KHz rotation, 1 s recycle, 56,976 repetitions) δ = − 104 (Q_3_), − 109 (Q_4_). Raman (solid, 532 nm, *ν*_max_/cm^−1^) 2960, 2899, 1252, 854, 726, 807, 726, 637, 423 and 385.

### Typical Ethylene Oligomerisation Procedure

A rigorously cleaned 150 mL stainless steel Parr autoclave (fitted with a pressure gauge, a bursting disk, and an internal K-type thermocouple) was taken into the glovebox under dynamic vacuum (~ 0.1 mbar) for 10 h. The vessel was charged with Cr{N(SiMe_3_)_2_}_x_/oxide (0.71 wt% Cr/oxide, 27 μmol Cr) and sealed under N_2_. The vessel was then connected to a Schlenk line and charged with a solution containing heptane (60 mL), nonane (1.0 mL) and MMAO-12 (7 wt% solution in toluene; 0.18 mL, 0.041 mmol) under a flow of N_2_ *via* a cannula. The autoclave was sealed under N_2_, before being purged with ethylene (1 mL s^−1^) for 10 s and then sealed. The contents of the reactor were cautiously heated to 120 °C using an external solid-state electrical band heater, whilst being agitated at 500 rpm using a customised magnetically-coupled overhead stirrer fitted with a turbine-type four-blade impeller. On reaching 120 °C the reactor was then pressurised with ethylene to 8 barg, prior to being isolated. After 30 min, the reaction vessel was cooled in a water/ice bath to 4 °C (30 min.) and then slowly vented inside a fume hood. An aliquot of the resulting liquid fraction was sampled, quenched with a 1:1 mixture of toluene and an aqueous solution of HCl (10% w/w). A sample of this organic phase was taken, filtered through a plug of cotton wool/Celite, before being analysed by GC-FID against the internal standard, nonane. Any residual white solid product (PE) was isolated *via* filtration, dried to constant weight at RT in air overnight (~ 10 h) and analysed using DSC.

## Electronic supplementary material

Below is the link to the electronic supplementary material.

Supplementary material 1 (DOCX 394 KB)

## References

[1] McGuinness DS (2011). Chem Rev.

[2] Speiser F, Braunstein P, Saussine W (2005). Acc Chem Res.

[3] Dixon JT, Green MJ, Hess FM, Morgan DH (2004). J Organomet Chem.

[4] Manyik RM, Walker WE, Wilson TP (1977). J Catal.

[5] Briggs JR (1987) Process for trimerization. US4668838

[6] Briggs JR (1989). J Chem Soc Chem Commun.

[7] Reagen WK, Conroy BK (1994) Chromium Compounds and Uses Thereof. US5198563A

[8] Freeman JW, Buster JL, Knudsen RD (1999) Olefin production. US005856257

[9] Lashier ME, Freeman JW, Knudsen RD (1996) Olefin production. US5543375

[10] Carter A, Cohen SA, Cooley NA, Murphy A, Scutt J, Wass DF (2002). Chem Commun.

[11] Wass DF (2002) Olefin trimerisation using a catalyst comprising a source of chromium, molybdenum or tungsten and a ligand containing at least one phosphorous, arsenic or antimony atom bound to at least one (hetero)hydrocarbyl group. US6800702B2

[12] Wright CMR, Williams TJ, Turner ZR, Buffet J-C, O’Hare D (2017). Inorg Chem Front.

[13] Varga V, Hodík T, Lamac M, Horacek M, Zukal A, Zilkova N, Pinkas WOP (2015). J Org Chem.

[14] Karbach FF, Severn JR, Duchateau R (2015). ACS Catal.

[15] Chen Y, Callens E, Abou-Hamad E, Merle N, White AJP, Taoufik M, Copéret C, Le Roux E, Basset JM (2012). Angew Chem Int Ed.

[16] Peulecke N, Müller BH, Peitz SE, Aluri BR, Rosenthal U, Wohl AI, Müller W, Al-Hazmi MH, Mosa FM (2010). Chem Catal Chem.

[17] Nenu CN, Weckhuysen BM (2005). J Chem Soc Chem Commun.

[18] Monoi T, Sasaki Y (2002). J Mol Catal A.

[19] Finiels A, Fajula F, Hulea V (2014). Catal Sci Technol.

[20] Bryliakov KP, Talsi EP (2012). Coord Chem Rev.

[21] Britovsek GJ, McGuinness DS (2016). Chem Eur J.

[22] McDyre L, Carter E, Cavell KJ, Murphy DM, Platts JA, Sampford K, Ward BD, Gabrielli WF, Hanton MJ, Smith DM (2011). Organometallics.

[23] McDyre LE, Hamilton T, Murphy DM, Cavell KJ, Gabrielli WF, Hanton MJ, Smith DM (2010). Dalton Trans.

[24] Thapa I, Gambarotta S, Korobkov I, Duchateau R, Kulangara SV, Chevalier R (2010). Organometallics.

[25] Brückner A, Jabor JK, McConnell AEC, Webb PB (2008). Organometallics.

[26] Tang S, Liu Z, Yan X, Li N, Cheng R, He X, Liu B (2014). Appl Catal A.

[27] McGuinness DS, Rucklidge AJ, Tooze RP, Slawin AMZ (2007). Organometallics.

[28] Agapie T, Labinger JA, Bercaw JE (2007). J Am Chem Soc.

[29] Blann K, Bollmann A, de Bod H, Dixon JT, Killian E, Nongodlwana P, Maumela MC, Maumela H, McConnell AE, Morgan DH, Overett MJ, Prétorius M, Kuhlmann S, Wasserscheid P (2007). J Catal.

[30] Chen EYX, Marks TJ (2000). Chem Rev.

[31] Ehm C, Cipullo R, Budzelaar PHM, Busico V (2016). Dalton Trans.

[32] Wright WRH, Batsanov AS, Howard JAK, Tooze RP, Hanton MJ, Dyer PW (2010). Dalton Trans.

[33] Jabri A, Mason CB, Sim Y, Gambarotta S, Burchell TJ, Duchateau R (2008). Angew Chem Int Ed.

[34] Vidyaratne I, Nikiforov GB, Gorelsky SI, Gambarotta S, Duchateau R, Korobkov I (2009). Angew Chem Int Ed.

[35] Hagimoto H, Shiono T, Ikeda T (2004). Macromol Chem Phys.

[36] Tanaka R, Kawahara T, Shinto Y, Nakayama Y, Shiono T (2017). Macromolecules.

[37] Kulangara SV, Haveman D, Vidjayacoumar B, Korobkov I, Gambarotta S, Duchateau R (2015). Organometallics.

[38] Angelescu E, Nicolau C, Simon Z (1966). J Am Chem Soc.

[39] Stavropoulos P, Bryson N, Youinou MT, Osborn JA (1990). Inorg Chem.

[40] Alyea EC, Bradley DC, Copperthwaite RG (1972). J Chem Soc, Dalton Trans.

[41] van Rensburg WJ, Grové C, Steynberg JP, Stark KB, Huyser JJ, Steynberg PJ (2004). Organometallics.

[42] Tomov AK, Gibson VC, Britovsek GJP, Long RJ, van Meurs M, Jones DJ, Tellmann KP, Chirinos JJ (2009). Organometallics.

[43] Overett MJ, Blann K, Bollmann A, Dixon JT, Haasbroek D, Killian E, Maumela H, McGuinness DS, Morgan DH (2005). J Am Chem Soc.

[44] Do LH, Labinger JA, Bercaw JE (2012). Organometallics.

[45] Zilbershtein TM, Kardash VA, Suvorova VV, Golovko AK (2014). Appl Catal A.

[46] Chudek JA, Hunter G, McQuire GW, Rochester CH, Smith TFS (1996). J Chem Soc Faraday Trans.

[47] Hartman JS, Sherriff BL (1991). J Phys Chem.

[48] Devreux F, Boilot JP, Chaput F, Sapoval B (1990). Phys Rev Lett.

[49] Appendix V-, Luchinat C, Parigi G, Ravera E (2017). Relaxation by dipolar interaction between two Spins A2 - Bertini, Ivano. Nmr of paramagnetic molecules (Second Edition).

[50] Bertini I, Luchinat C, Parigi G, Ravera E (2017). Chap. 2 - the hyperfine shift. Nmr of Paramagnetic Molecules (Second Edition).

[51] Vansant EF, Voort PVD, Vrancken KC (1995) Chapter 5 the distribution of the silanol types and their desorption energies, In: Vansant EF, Voort PVD, Vrancken KC (eds) Studies in surface science and catalysis, vol 93. Elsevier, pp 93–126

[52] Zhuravlev LT (2000). Colloids Surf A.

[53] Vansant EF, Voort PVD, Vrancken KC, Vansant EF, Voort PVD, Vrancken KC (1995). Chapter 3 the surface chemistry of silica. Studies in surface science and catalysis.

[54] Monoi T, Ikeda H, Ohira H, Sasaki Y (2002). Polym J.

[55] Cossee P (1964). J Catal.

[56] Arlman EJ (1964). J Catal.

[57] Arlman EJ, Cossee P (1964). J Catal.

[58] Brown C, Krzystek J, Achey R, Lita A, Fu R, Meulenberg RW, Polinski M, Peek N, Wang Y, Burgt LJ, Profeta S, Stiegman AE, Scott SL (2015). ACS Catal.

[59] McDaniel MP (2010). Adv Catal.

[60] Debecker DP, Stoyanova M, Rodemerck U, Léonard A, Su BL, Gaigneaux EM (2011). Catal Today.

[61] Edwards DN, Briggs JR, Marcinkowsky AE, Lee KH (1988) Process for the preparation of aluminoxanes. US4772736

[62] Bradley DC, Copperthwaite RG, Extrine MW, Reichert WW, Chisholm MH (1972). J Chem Soc Dalton Trans.

