# Supplementary material for: The Role of Catalyst Support, Diluent and Co-Catalyst in Chromium-Mediated Heterogeneous Ethylene Trimerisation
Source: Top Catal. 2018 Jan 16;61(3):213–24. doi: 10.1007/s11244-018-0891-8 (PMC6413812; doi:10.1007/s11244-018-0891-8)
Supplement: Supplementary file 1 — Supplementary material 1 (DOCX 394 KB) [file 11244_2018_891_MOESM1_ESM.docx]

*Topics in Catalysis* – **Supplementary Information**

**Role of Catalyst Support, Solvent and Co-Catalyst in Chromium-mediated Heterogeneous Ethylene Trimerisation**

M. J. Lamb,^1,2^ D. C. Apperley,^1^ M. Watson,^3^ P. W. Dyer^1,2^*

^1^ Department of Chemistry, Durham University, South Road, Durham, DH1 3LE

^2^ Centre for Sustainable Chemical Processes, Department of Chemistry, Durham University, South Road, Durham, DH1 3LE

^3^ Johnson Matthey PLC, PO Box 1, Billingham, Cleveland, TS23 1LB

P. W. Dyer

p.w.dyer@durham.ac.uk; +44(0)191 334 2150

ORCiD: orcid.org/0000-0001-6576-5619

**
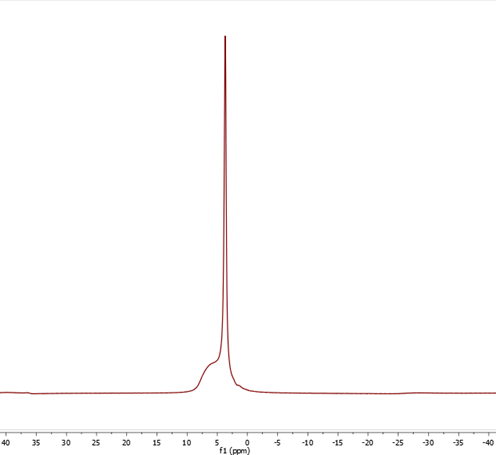
**

S1 Solid-state direct excitation magic-angle spinning ^1^H NMR spectrum of as-received Evonik Aeroperl 300/30 fumed silica; 400 MHz spectrometer frequency, 13 KHz spin-rate.


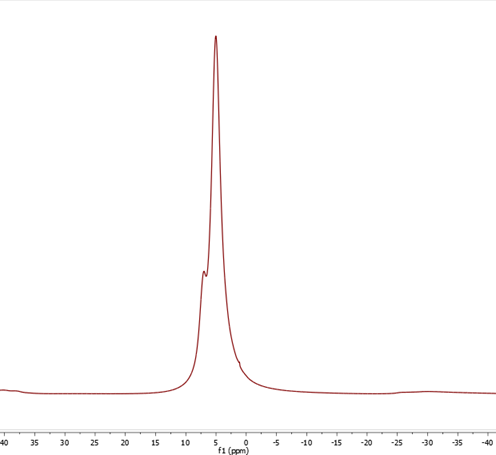


S2 Solid-state direct excitation magic-angle spinning ^1^H NMR spectrum of as-received Sigma Aldrich silica-alumina grade 135 catalyst support; 400 MHz spectrometer frequency, 13 KHz spin-rate.


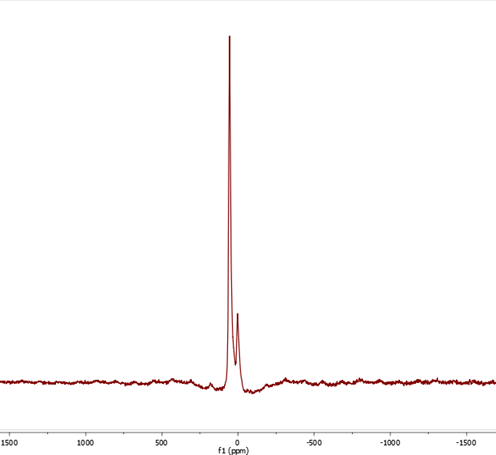


S3 Solid-state direct excitation magic-angle spinning ^27^Al NMR spectrum of as-received Sigma Aldrich silica-alumina grade 135 catalyst support; 104 MHz spectrometer frequency, 13 KHz spin-rate.


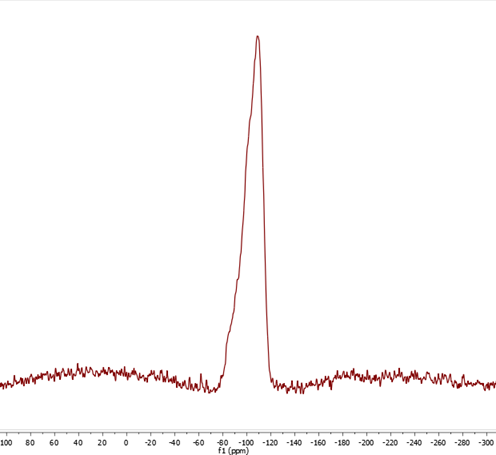


S4 Solid-state direct excitation ^29^Si NMR spectrum of as-received Sigma Aldrich silica-alumina grade 135 catalyst support; 79 MHz spectrometer frequency, 6 KHz spin-rate.


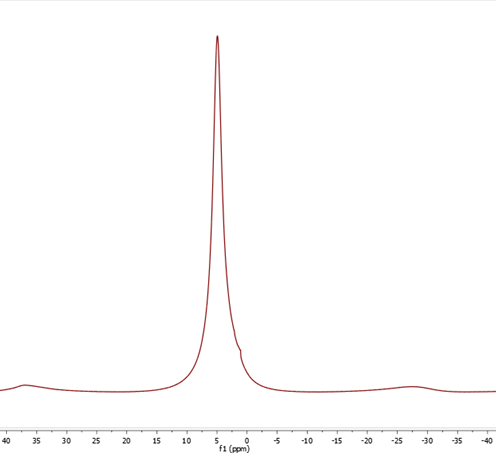


S5 Solid-state direct excitation magic-angle spinning ^1^H NMR spectrum of as-received Alfa Aesar γ-alumina (1/8” pellets ground and sieved to < 250 μm); 400 MHz spectrometer frequency, 13 KHz spin-rate.


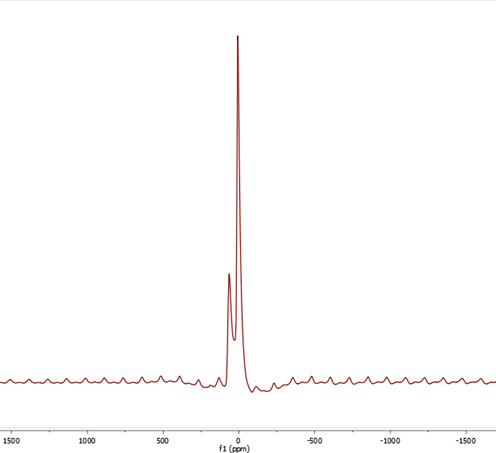


S6 Solid-state direct excitation magic-angle spinning ^27^Al NMR spectrum of as-received Alfa Aesar γ-alumina (1/8” pellets ground and sieved to < 250 μm); 104 MHz spectrometer frequency, 13 KHz spin-rate.

S7 ICP-OES Trace metal analyses of as-received Evonik Aeroperl 300/30 fumed silica, Sigma Aldrich silica-alumina grade 135 catalyst support and Alfa Aesar γ-alumina (1/8” pellets ground and sieved to < 250 μm)


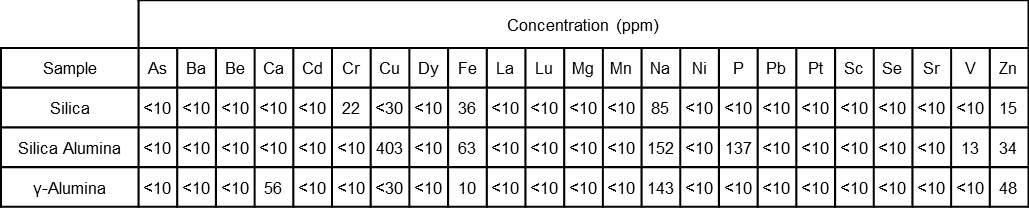


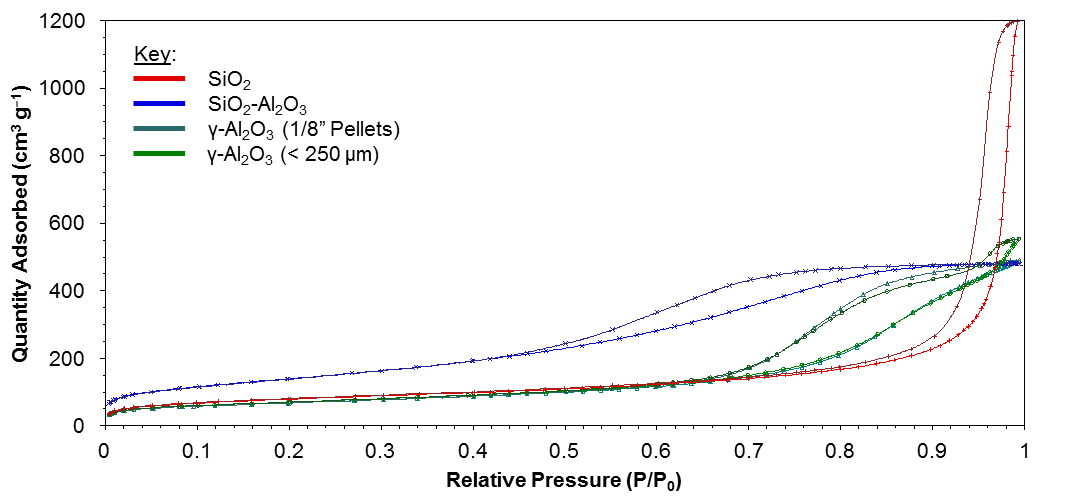


S8 BET isotherm linear plot of as-received Evonik Aeroperl 300/30 fumed silica, Sigma Aldrich silica-alumina grade 135 catalyst support, Alfa Aesar γ-alumina (1/8” pellets) and Alfa Aesar γ-alumina (1/8” pellets ground and sieved to < 250 μm)

**
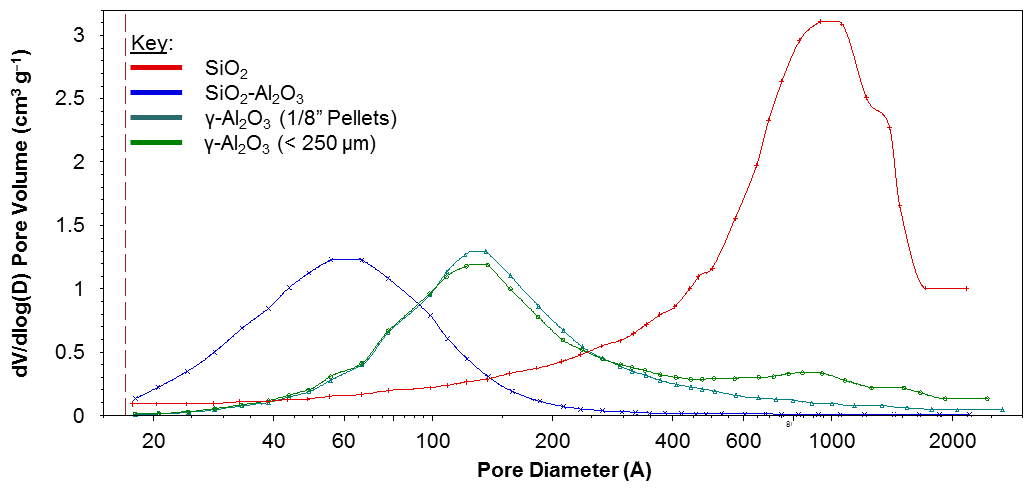
**

S9 BJH adsorption pore volume plot of as-received Evonik Aeroperl 300/30 fumed silica, Sigma Aldrich silica-alumina grade 135 catalyst support, Alfa Aesar γ-alumina (1/8” pellets) and Alfa Aesar γ-alumina (1/8” pellets ground and sieved to < 250 μm)


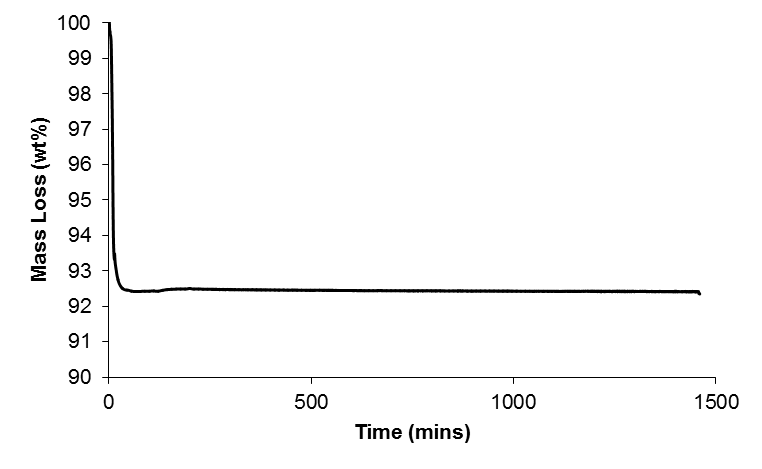


S10 TGA of as-received Evonik Aeroperl 300/30 fumed silica from 30 – 600 °C, at a ramp rate of 30 °C min^–1^

**
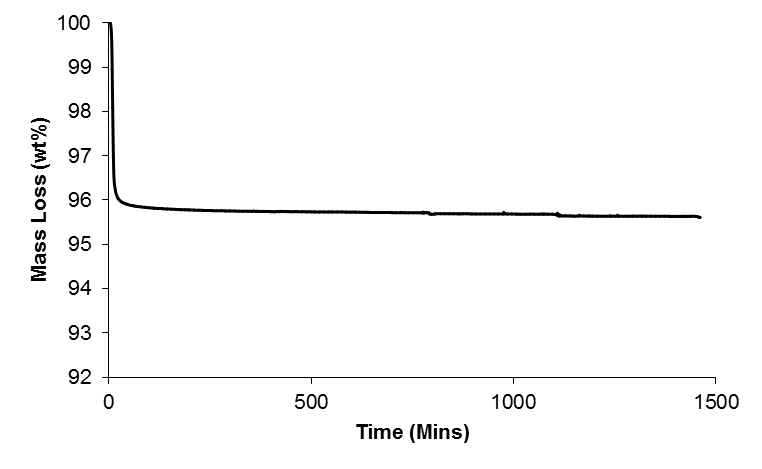
**

S11 TGA of as-received Sigma Aldrich silica-alumina grade 135 catalyst support from 30 – 600 °C, at a ramp rate of 30 °C min^–1^

**
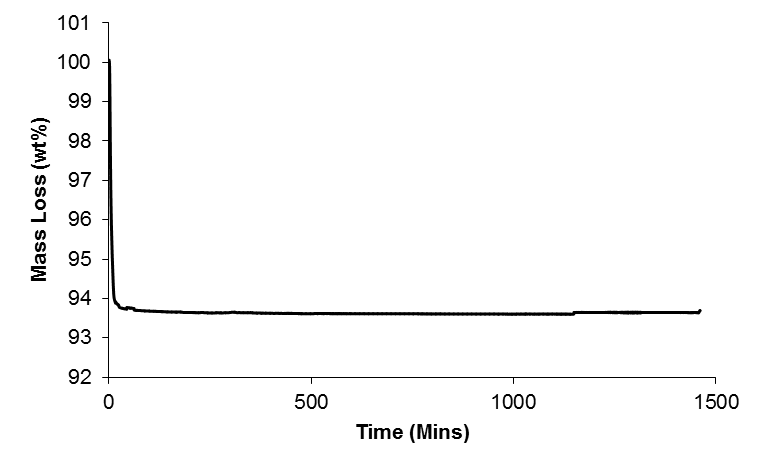
**

S12 TGA of as-received Alfa Aesar γ-alumina (1/8” pellets ground and sieved to < 250 μm) from 30 – 600 °C, at a ramp rate of 30 °C min^–1^


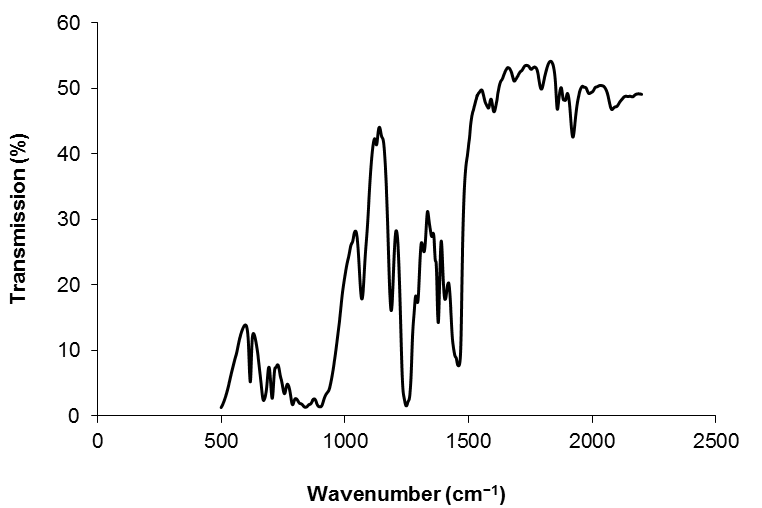


S13 IR (KBr, Nujol *ν*_max_/cm^–1^) spectrum of Cr{N(SiMe_3_)_2_}_3_


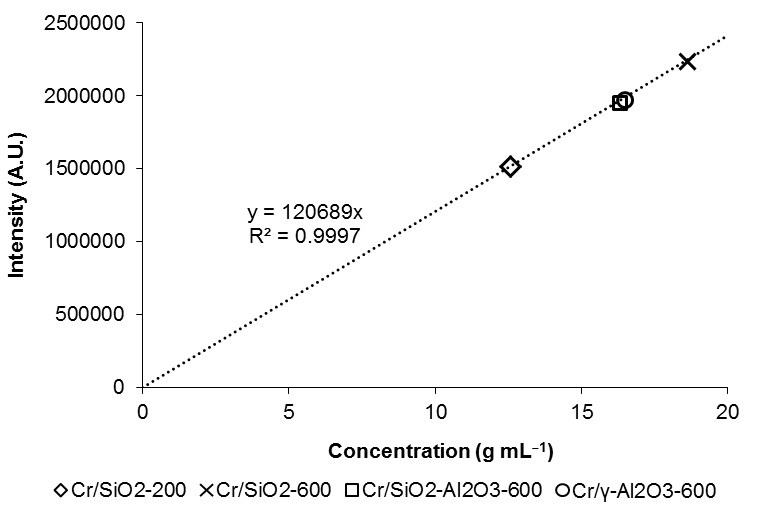


S14 ICP-OES chromium metal analyses (357.9 nm): (a) Cr{N(SiMe_3_)_2_}_x_/SiO_2-200_; (b) Cr{N(SiMe_3_)_2_}_x_/SiO_2-600_; (c) Cr{N(SiMe_3_)_2_}_x_/SiO_2_-Al_2_O_3-600_; (d) Cr{N(SiMe_3_)_2_}_x_/γ-Al_2_O_3-600_
